# First results of the German Barcode of Life (GBOL) – Myriapoda project: Cryptic lineages in German *Stenotaenia linearis* (Koch, 1835) (Chilopoda, Geophilomorpha)

**DOI:** 10.3897/zookeys.510.8852

**Published:** 2015-06-30

**Authors:** Thomas Wesener, Karin Voigtländer, Peter Decker, Jan Philip Oeyen, Jörg Spelda, Norman Lindner

**Affiliations:** 1 Zoologisches Forschungsmuseum Alexander Koenig, Leibniz Institute for Animal Biodiversity, Center for Taxonomy and Evolutionary Research (Section Myriapoda), Adenauerallee 160, 53113 Bonn, Germany; 2 Senckenberg Museum of Natural History Görlitz, Am Museum 1, 02826 Görlitz, Germany; 3 Bavarian State Collection of Zoology, Münchhausenstraße 21, 81247 Munich, Germany; 4 Lazarusstraße 34, 04347 Leipzig, Germany

**Keywords:** Barcode, biodiversity, COI, cryptic diversity

## Abstract

As part of the German Barcode of Life (GBOL) Myriapoda program, which aims to sequence the COI barcoding fragment for 2000 specimens of Germany’s 200 myriapod species in the near future, 44 sequences of the centipede order Geophilomorpha are analyzed. The analyses are limited to the genera *Geophilus* Leach, 1814 and *Stenotaenia* Koch, 1847 and include a total of six species. A special focus is *Stenotaenia*, of which 19 specimens from southern, western and eastern Germany could be successfully sequenced. The *Stenotaenia* data shows the presence of three to four vastly different (13.7–16.7% p-distance) lineages of the genus in Germany. At least two of the three lineages show a wide distribution across Germany, only the lineage including topotypes of *Stenotaenia linearis* shows a more restricted distribution in southern Germany. In a maximum likelihood phylogenetic analysis the Italian species Stenotaenia ‘sorrentina’ (Attems, 1903) groups with the different German *Stenotaenia linearis* clades. The strongly different *Stenotaenia linearis* lineages within Germany, independent of geography, are a strong hint for the presence of additional, cryptic *Stenotaenia* species in Germany.

## Introduction

The German Barcode of Life – Myriapoda project aims to sequence part of the mitochondrial cytochrome *c* oxidase subunit I gene known as the barcode fragment for all approximately 200 Myriapoda species in Germany (Voigtländer et al. 2011). Introduced species, mainly from greenhouses (Decker et al. 2014), will also be included. Myriapod barcoding is still in its infancy. While some studies incorporate COI data, this is mostly done on the species-level (e.g. Oeyen et al. 2014), and occasionally in genus-level studies (e.g. Stoev et al. 2010, Wesener et al. 2014). In Germany, a study of Bavarian myriapods (Spelda et al. 2011) pioneered research in this field.

Here, we show the preliminary results of one of the largest barcoding datasets compiled for centipedes of the order Geophilomorpha, with a special focus on the recently revised *Stenotaenia* Koch, 1847 (Bonato and Minelli 2008). *Stenotaenia* is distributed in Europe and the adjacent Mediterranean area and now includes 15 valid species. *Stenotaenia linearis* (Koch, 1835) is the type species of the genus, and the only species recorded from Germany (Voigtländer et al. 2011). After the resurrection of the genus in 2008, some redescriptions were undertaken (Dányi 2010), and the species *Stenotaenia linearis* was recorded from Belgium for the first time (Lock 2009).

The taxonomic situation of the type species of *Stenotaenia*, *Stenotaenia linearis*, is slightly confused, as the original Koch type specimens from Regensburg, Germany are apparently lost (Bonato and Minelli 2008). Seven species are currently synonymized under the name *Stenotaenia linearis* (Bonato & Minelli, 2014). Another four valid species, *Stenotaenia asiaeminoris* (Verhoeff, 1898), *Stenotaenia giljarovi* (Folkmanova, 1956), *Stenotaenia naxia* (Verhoeff, 1901), and *Stenotaenia palaestina* (Verhoeff, 1925), spanning the entire geographical range of the genus, are difficult to distinguish from *Stenotaenia linearis* (see Bonato and Minelli 2008). A correct definition of *Stenotaenia linearis* is therefore a crucial necessity for any further taxonomic work in the genus.

Molecularly, little was done in *Stenotaenia*. One specimen of *Stenotaenia linearis* was used for the Fauna Bavarica project (Spelda et al. 2011). Of other *Stenotaenia* species, only one sequence of Stenotaenia ‘sorrentina’ (Attems, 1903), a putative synonym (ICZN 2014) of *Geophilus forficularius* Fanzago, 1881, which was part of a recent phylogenetic study (Bonato et al. 2014) can be found. The discovery of unusually large genetic distances between different clades in German *Stenotaenia linearis*, not found in any other German Geophilomorpha, and potentially independent of biogeography, prompted us to focus our attention on this species. In this study, the genetic distances in between German *Stenotaenia linearis* specimens are geographically analyzed and interpreted.

## Material and methods

### Specimen collection and preparation

Specimens were determined and collected by the authors of the study by hand, and either directly or after a few days transferred to vials containing 95% undenatured ethanol. The vials contain an individual GBOL number with which the specimens can be connected to the accompanying data. After conservation the specimens were either sent to the GBOL facility at the Museum Koenig, Bonn, Germany (ZFMK) or to the corresponding laboratory at the Bavarian State collection of Zoology, Munich, Germany (ZSM). Upon arrival, all specimens were photographed (images will be uploaded to BOLD, http://www.boldsystems.org/), and a tissue sample was removed for DNA extraction. All specimens will later be stored as vouchers in 95% undenatured ethanol, either at the ZFMK, the SMNG (Senckenberg Museum für Naturkunde, Görlitz) or the ZSM (see Table 1). For this specific GBOL subproject, DNA extraction was attempted for more than 35 specimens of *Geophilus* and 24 *Stenotaenia*, all specimens from Germany.

**Table 1.** GBOL numbers, Genbank codes, locality data. GBOL number refers to DNA extraction and BOLD registration. SMNG = Senckenberg Museum für Naturkunde, Görlitz, Germany; ZFMK = Zoological Research Museum A. Koenig, Bonn, Germany; ZSM = Zoologische Staatssammlung München, Germany.

GBOL	GenBank	Voucher	Species	Locality
ZFMK-TIS-1318	KM999124	SMNG VNR016755-1	*Geophilus alpinus*	Saxony, Hirschfelde, Neißetal.
ZFMK-TIS-1449	KM999119	ZFMK MYR3840	*Geophilus alpinus*	Saxony, Leipzig, Leipziger Auwald, Revierort „Die Nonne“
ZFMK-TIS-1520	KM999120	ZFMK MYR3871	*Geophilus alpinus*	Saxony, Jähstadt, Annaberger Ratswald.
ZFMK-TIS-1560	KM999118	ZFMK MYR3875	*Geophilus alpinus*	Bavaria, Donaustauf.
ZFMK-TIS-1647	KM491674	ZFMK MYR3720	*Geophilus alpinus*	Saxony-Anhalt, Ilsenburg, Ilsetal.
ZFMK-TIS-1656	KM491579	ZFMK MYR3725	*Geophilus carpophagus*	Saxony-Anhalt, Ilsenburg, Ilsensteinhang.
ZFMK-TIS-2519834	KM491622	ZFMK MYR3813	*Geophilus carpophagus*	Saxony-Anhalt, Ilsenburg, Ilsensteinhang.
ZFMK-TIS-1413	KM491587	ZFMK MYR3653	*Geophilus electricus*	Saxony-Anhalt, Nordharz, Heudeber.
ZFMK-TIS-1518	KM491687	ZFMK MYR3673	*Geophilus electricus*	Saxony-Anhalt, Halberstadt, Athenstedt.
ZFMK-TIS-1650	KM491673	ZFMK MYR3723	*Geophilus electricus*	Saxony-Anhalt, Ilsenburg, Dreisageblocksberg.
ZFMK-TIS-19414	KM491636	ZFMK MYR2107	*Geophilus electricus*	North Rhine-Westphalia, Windeck, Stromberg.
ZFMK-TIS-1468	KM999123	ZFMK MYR3850	*Geophilus flavus*	Saxony, Zwickau, Brückeberg.
ZFMK-TIS-1525	KM491642	ZFMK MYR3676	*Geophilus flavus*	Saxony-Anhalt, Schönhausen (Elbe).
ZFMK-TIS-1603	KM491670	ZFMK MYR3705	*Geophilus flavus*	Saxony-Anhalt, Gerbstedt, Friedeburg.
ZFMK-TIS-6359	KM491617	ZFMK MYR3536	*Geophilus flavus*	Saxony, Gröditz, Weißenberg.
ZFMK-TIS-15516	KM491627	ZFMK MYR1004	*Geophilus flavus*	North Rhine-Westphalia, Bonn, Oberkassel Steinbruch.
ZFMK-TIS-15764	KM491602	ZFMK MYR1060	*Geophilus flavus*	North Rhine-Westphalia, Wuppertal, NSG ‚Im Hölken‘
ZFMK-TIS-15774	KM491626	ZFMK MYR1070	*Geophilus flavus*	North Rhine-Westphalia, Siebengebirge, Löwenburg.
ZFMK-TIS-15821	KM491693	ZFMK MYR1117	*Geophilus flavus*	North Rhine-Westphalia, Wuppertal, Dolinengelände Krutscheid.
ZFMK-TIS-19577	KM491685	ZFMK MYR1526	*Geophilus flavus*	North Rhine-Westphalia, Bonn, Kottenforst.
ZFMK-TIS-19591	KM491632	ZFMK MYR1543	*Geophilus flavus*	North Rhine-Westphalia, Heimbach, Meuchelberg.
ZFMK-TIS-19602	KM491649	ZFMK MYR1554	*Geophilus flavus*	North Rhine-Westphalia, Königswinter, Südhang Wolkenburg.
ZFMK-DNA-112780112	KM491570	ZSM-JSP100815-007	*Geophilus flavus*	North Rhine-Westphalia, Bielefeld, Brackweder Wald.
ZFMK-DNA-112780116	KM999125	ZSM-JSP120413-004	*Geophilus flavus*	Baden-Württemberg, Bad Urach, St. Johann Fohlenhof.
ZFMK-DNA-112780042	KM999126	ZSM-JSP120413-002	*Geophilus ribauti*	Baden-Württemberg, Bad Urach, St. Johann Fohlenhof.
ZFMK-TIS-19495	KM999122	ZFMK MYR1630	*Stenotaenia linearis*	North Rhine-Westphalia, Bonn, Oberkassel Steinbruch.
ZFMK-TIS-1450	KM999121	ZFMK-TIS-1450	*Stenotaenia linearis*	Saxony, Leipzig, Leipziger Auwald, Revierort „Die Nonne“.
ZFMK-TIS-15771	KM491663	ZFMK MYR1067	*Stenotaenia linearis*	North Rhine-Westphalia, Wuppertal, NSG ‚Im Hölken‘.
ZFMK-TIS-15861	KM491574	ZFMK MYR1157	*Stenotaenia linearis*	North Rhine-Westphalia, Wuppertal, Dolinengelände Krutscheid.
ZFMK-TIS-19430	KM491573	ZFMK MYR2030	*Stenotaenia linearis*	Rheinland-Pfalz, Altenkirchen, Seelbach bei Hamm.
ZFMK-DNA-112780045	KM491689	ZSM-JSP120412-003	*Stenotaenia linearis*	Baden-Württemberg, Esslingen, St. Bernhard.
ZFMK-DNA-112780062	KM491558	ZSM-JSP100514-021	*Stenotaenia linearis*	Bavaria, Dachau, palace garden.
ZFMK-DNA-112780066	KM491631	ZSM-JSP120411-001	*Stenotaenia linearis*	Baden-Württemberg, Esslingen, St. Bernhard.
ZFMK-DNA-112780069	KM491658	ZSM-JSP120408-007	*Stenotaenia linearis*	Baden-Württemberg, Hegnach, Hardtwald.
ZFMK-DNA-112780093	KM491637	ZSM-JSP120408-002	*Stenotaenia linearis*	Baden-Württemberg, Stuttgart, SW Max-Eyth-See.
GBOL11002	KP698104	ZSM-JSP141102-010	*Stenotaenia linearis*	Bavaria, Regensburg
GBOL10999	KP698105	ZSM-JSP141102-004	*Stenotaenia linearis*	Bavaria, Regensburg
ZFMK-TIS-19423	KR559681	ZFMK MYR2119	*Stenotaenia linearis*	North Rhine-Westphalia, Euskirchen, Bad Münstereifel, Gilsdorf.
ZFMK-TIS-2538216	KR559680	ZFMK MYR3467	*Stenotaenia linearis*	Saxony, Dresden, Gruna
ZFMK-TIS-1645	KR559679	ZFMK MYR3878	*Stenotaenia linearis*	Saxony, Zwickau, Brueckeberg
GBOL12266	KR736251	SMNG-VNR016704-1	*Stenotaenia linearis*	North Rhine-Westphalia. Bochum, Tippelsberg
GBOL12450	KR736248	ZSM-JSP150117-056	*Stenotaenia linearis*	Baden-Württemberg, Breisgau, Badenweiler
GBOL12421	KR736250	SMNG-MYR016705-1	*Stenotaenia linearis*	North Rhine-Westphalia. Unna, Selm
GBOL11224	KR736249	ZSM-JSP141113-005	*Stenotaenia linearis*	Baden-Württemberg, Ulm, Kiesental

### DNA extraction and sequencing

At the ZFMK, DNA was extracted from the tissue samples using the BioSprint96 magnetic bead extractor by Qiagen (Germany). After the extraction, samples were outsourced for PCR and sequencing (BGI China). For PCR and sequencing, HCO/LCO primer pairs (Folmer et al. 1994) were utilized. Because of a low PCR and sequencing success (<50%) for the Myriapoda, the degenerated primer pair HCOJJ/LCOJJ (Astrin and Stüben 2008) was used for further sequencing attempts, resulting in a much higher success rate (>75%). At the ZSM, a tissue sample was removed from each specimen and transferred into 96 well plates for subsequent DNA extraction at the Canadian Center for DNA Barcoding (CCDB) where they were processed using standard barcoding protocols. All protocols for DNA extraction, PCR amplifications and Sanger Sequencing procedures are available online under: http://www.dnabarcoding.ca/pa/ge/research/protocols.for DNA. DNA was extracted from the whole voucher at the CCDB. All samples were PCR amplified with modified Folmer primers CLepFolF, and the same primers were employed for subsequent Sanger sequencing. All voucher information and the DNA barcode sequences, primer pairs and trace files were uploaded to BOLD (http://www.boldsystems.org).

However, for more than five *Stenotaenia linearis* and more than 10 *Geophilus* specimens no sequences could be obtained. Sequences were obtained for 19 *Stenotaenia* and 25 *Geophilus* specimens. Sequence identities were confirmed with BLAST searches (Altschul et al. 1997). All 44 new sequences were deposited in GenBank (see Table 1 for accession numbers). The only available COI sequence of *Stenotaenia* (KF569300.1), labelled as *Stenotaenia sorrentina*, was added to the dataset. In order to rule-out the accidental amplification of nuclear copies of the mitochondrial COI gene, the whole dataset was translated into amino acids following the ‘invertebrate’ code in MEGA6 (Tamura et al. 2013); internal stop codons were absent in our dataset. There were a total of 658 positions in the final dataset, gaps were absent.

### Phylogenetic analysis

Sequences were aligned by hand in Bioedit (Hall 1999). The final dataset included 45 nucleotide sequences with 658 positions (44 newly sequenced and the one of Stenotaenia ‘sorrentina’ from GenBank). Phylogenetic analyses were conducted in MEGA6 (Tamura et al. 2013). A Modeltest, as implemented in MEGA6 (Tamura et al. 2013), was performed to find the best fitting maximum likelihood substitution model. Models with the lowest BIC scores (Bayesian Information Criterion) are considered to describe the best substitution pattern. Codon positions included were 1st+2nd+3rd+Noncoding. Modeltest selected the Tamura-Nei model (Tamura and Nei 1993) with gamma distribution and invariant sites as best fitting model (lnL -4245.19958, Invariant 0.55674, Gamma 1.176355, R 3.46, Freq A: 0.288843, T: 0.282885, C: 0.262778, G: 0.16546).

The evolutionary history was inferred by using the maximum likelihood method based on the selected Tamura-Nei model (Tamura and Nei 1993). The tree with the highest log likelihood (-4247.0145) is shown (Nei and Kumar 2000). The percentage of trees in which the associated taxa clustered together is shown next to the branches. Initial tree(s) for the heuristic search were obtained automatically by applying Neighbor-Join and BioNJ algorithms to a matrix of pairwise distances estimated using the Maximum Composite Likelihood (MCL) approach, and then selecting the topology with superior log likelihood value. A discrete Gamma distribution was used to model evolutionary rate differences among sites (5 categories (+G, parameter = 1.1347)). The rate variation model allowed for some sites to be evolutionarily invariable ([+I], 55.5093% sites). The tree is drawn to scale, with branch lengths measured in the number of substitutions per site.

### Distance analysis

The number of pairwise base differences per site were calculated in MEGA6 (Tamura et al. 2013). Codon positions included were 1st+2nd+3rd+Noncoding. In the distance analysis, all positions containing ‘N’s were removed for each sequenced pair. There were a total of 658 positions in the final dataset. To further evaluate the divergence within the genera *Geophilus* and *Stenotaenia*, the frequency distribution of the pairwise intra- and inter-specific distances were analysed.

## Results

### Phylogenetic analysis

**Figure 1.**
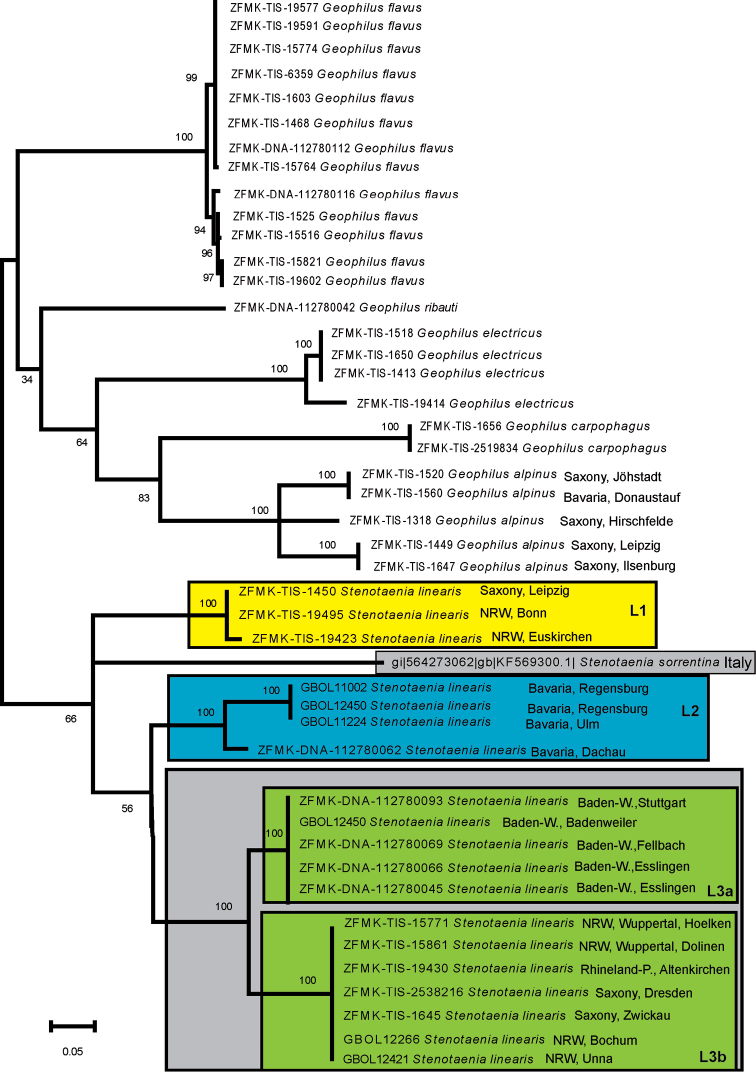
Maximum likelihood tree, 1000 bootstrap replicates. L1–L3 = *Stenotaenia linearis* lineages 1–3; NRW = North Rhine-Westphalia; Baden-W = Baden-Württemberg. Stenotaenia ‘sorrentina’ comes from GenBank and might refer to *Stenotaenia forficularis*. For exact locality data, see Table 1.

*Geophilus* is not clearly separated from *Stenotaenia* in our analysis (Fig. 1). The basal-most node of the tree supports three monophyletic groups: *Geophilus flavus* (de Geer, 1778), a species formerly separated in a different genus, *Necrophloeophagus* Newport, 1842, all other *Geophilus*, and *Stenotaenia*. However, the other *Geophilus* receive little statistical support (34%). The monophyly of the individual *Geophilus* species, as well as the *Stenotaenia* lineages L1–L3, all receive 100% bootstrap support (Fig. 1).

All 13 specimens of *Geophilus flavus* show little genetic distance (0–2.4%) to one another. Within the group containing the remaining *Geophilus* species, *Geophilus ribauti* Brölemann, 1908, a species formerly treated as a member of the genus *Brachygeophilus* Brölemann, 1908, is in a basal position to a weakly supported clade (64% statistical support) including *Geophilus electricus* (Linné, 1758), *Geophilus carpophagus* Leach, 1814, and *Geophilus alpinus* Meinert, 1870. In this clade, *Geophilus electricus* (100% statistical support) is opposed to the sister-taxa *Geophilus carpophagus* and *Geophilus alpinus* (83% statistical support). Inside *Geophilus electricus*, the one specimen from western Germany is opposed to the three from Saxony-Anhalt (Table 1 and Fig. 1). *Geophilus alpinus* is the only analyzed *Geophilus* species with widely separated intraspecific groups (Fig. 1). A basal trichotomy (Fig. 1) divides the five analyzed specimens into three groups that can not be separated geographically.

Within *Stenotaenia*, a basal trichotomy separates the specimens into (1) *Stenotaenia linearis* L1, (2) Stenotaenia ‘sorrentina’, and (3) the weakly supported (56% bootstrap support) *Stenotaenia linearis* L2 (including the topotypes) together with *Stenotaenia linearis* L3 (Fig. 1). *Stenotaenia linearis* L1 includes three specimens, one from Bonn, another from Euskirchen, both in western Germany and one from Leipzig in eastern Germany. *Stenotaenia linearis* L2 contains a single specimen from Dachau, one close to Ulm, as well as two topotypes from Regensburg, all in southern Germany, while the majority (12) of analyzed German *Stenotaenia linearis* specimens are recovered in *Stenotaenia linearis* L3 (Fig. 1). The L3 group is divided into two clusters (L3a and b), one including seven specimens representing a single haplotype from seven different localities in western and eastern Germany, and the other one including five specimens also representing a single haplotype from four different localities (Esslingen, Hegnach, Badenweiler, and Stuttgart) in south-western Germany.

### Distance analysis

**Figure 2.**
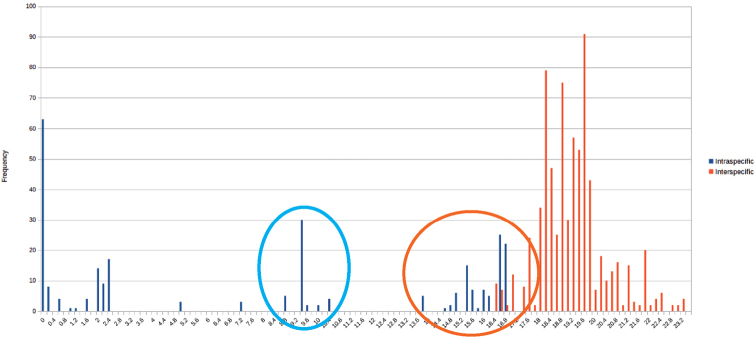
Frequency distribution of pairwise intraspecific (blue) and interspecific (red) distances. Blue circle = intraspecific distances of *Geophilus alpinus* and among *Stenotaenia linearis* L3; Red circle = interspecific distances and distances between *Stenotaenia linearis* lineages. Basic table see Suppl. material 1.

The distance analysis shows a first cluster of intraspecific distances ranging from 0–2.8%, with a *Geophilus electricus* outlier at 4.9% (Fig. 2), a second cluster at 9.4–10.2%, and a third cluster, which overlaps with the interspecific distances, at 13.7–16.7%. Interspecific distances inside German *Geophilus* and *Stenotaenia* are high, varying from 16.3–22.0%. The highest observed genetic distance is between *Stenotaenia* and *Geophilus* species (16.6–22.7%), while the *Geophilus* species differ from one another by 17.2–21.7%.

## Discussion

### Distance analysis

Clear intraspecific distances in German Geophilomorpha range from 0–5% (Fig. 2). A potential barcoding gap, however, is filled by the relatively high intraspecific distances (Fig. 2) of *Geophilus alpinus* and *Stenotaenia* L3 (9.4–10.2% range). The genetic distances (13.7–16.7%) between the different *Stenotaenia* lineages (L1, L2 & L3) fall partly in the interspecific range of variation of the German Geophilomorpha (Fig. 2). The large interspecific distances (16.6–22.7%) observed among German Geophilomorpha are an indication that all species can be easily separated using the COI barcode marker. The distance analysis is partly biased towards interspecific distances because only a few specimens per species were analyzed. To explain the high nucleotide variability, excluding cryptic species, the presence of the maternally inherited endosymbionts (Hurst et al. 2005), as well as the origin of the lineages from different glacial refugia (Babik et al. 2005) followed by a subsequent fusion to a single species, need to be checked.

### Three lineages of *Stenotaenia* in Germany

**Figure 3.**
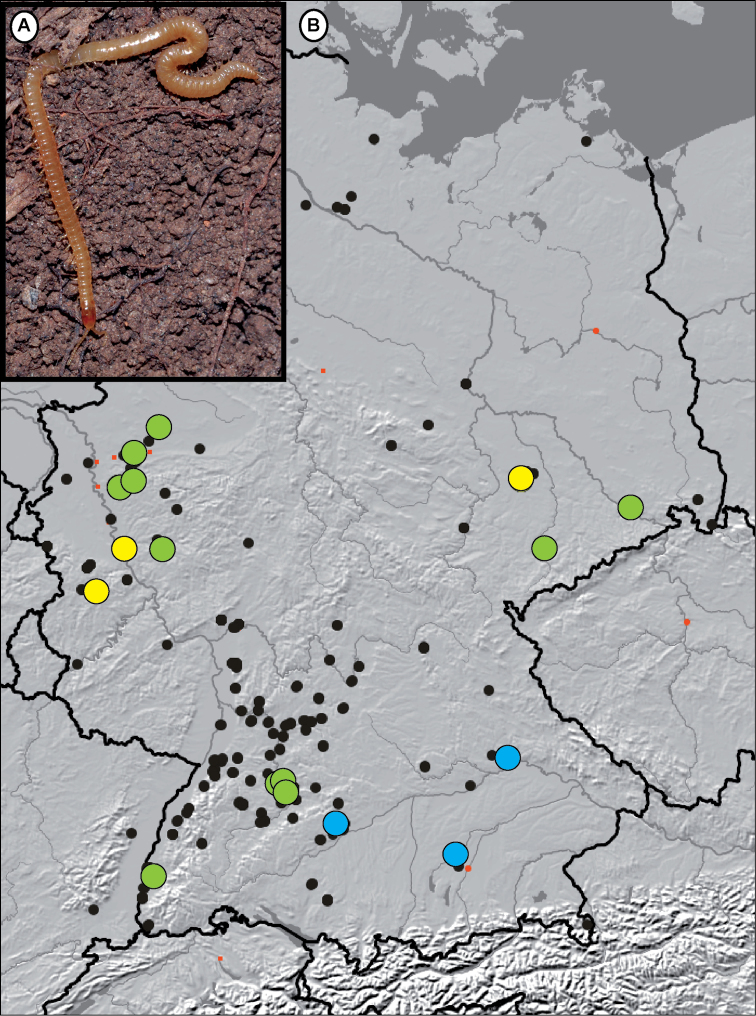
Map of *Stenotaenia linearis* samples studied during GBOL (large dots), as well as other *Stenotaenia linearis* records from Edaphobase, the ZSM and ZFMK collection (small dots, status 10.2014). Yellow = *Stenotaenia linearis* L1; Blue = *Stenotaenia linearis* L2; Green = *Stenotaenia linearis* L3. (A) *Stenotaenia linearis* in the field, photo: J. Spelda, specimen from Stuttgart-Hofen, Zuckerberg.

The three German *Stenotaenia* lineages are only weakly geographically separated (Fig. 3). *Stenotaenia* L1 is represented in our dataset with one specimen from Bonn, one from Euskirchen, and a third specimen from Leipzig (Fig. 1), the first two localities are separated from the third by more than 400 km apart (Fig. 3). This clade can be described as the *Stenotaenia* specimen from central Germany. All three specimens show the same haplotype.

*Stenotaenia* L2 represents topotypic material from Regensburg, a specimen from the Kiesental near Ulm, as well as a single specimen from Dachau in southern Germany. All three localities are more than 100 km apart but only the specimen from Dachau differs by 1.4%. *Stenotaenia* L2 differs significantly (13.7–16.7%) from other German *Stenotaenia*. This clade might be characterised as of southeastern German origin along the Danube river system.

Both clades of lineage 3, one from western and eastern Germany (L3a), the other from SW Germany (L3b) show identical haplotypes, but differ from one another by 9.4% (Fig. 3). The intraspecific difference is similar to the differences observed in some *Geophilus* species (9.4–10.2% in *Geophilus alpinus*), but significantly larger than the differences observed in the widespread *Geophilus flavus* (0.2–2.4%), which often come from the exact same localities as the *Stenotaenia* specimens (Table 1).

Whether or not the apparent sympatric distribution of the three different lineages of *Stenotaenia* in Germany (Fig. 3) might have been influenced by human-induced introduction or dispersal is not known. Virtually all collection localities are close to human habitats, but differ strongly in their current direct exposure to human activities.

### Potential analysis problems and what we can learn for future work

Such a large project faces a set of predictable technical problems, which can potentially cause wrong results.

Specimen collections: According to the main aim of the project (get approx. 10 specimens from at least five localities for each species to capture the estimated German-wide COI variation), the different collectors preferred localities where they could find many myriapod specimens easily – a potential collection bias. The amount of successfully sequenced *Stenotaenia linearis* specimens as well as *Geophilus alpinus* specimens and their different positions and deep splits within the maximum likelihood tree (Fig. 1) tell us that a larger amount of specimens from many more regions in Germany (Fig. 3) would be a desirable object for future taxonomic and/or biogeographical studies on these species. Bergsten et al. (2012) showed that up to 70 individuals are required to sample 95% of the intraspecific variation.

Specimen determination: As done by Bonato et al. (2014) for all Geophilomorpha species, a data matrix of additional morphological characters, presumably morphometric characters, should be created for the detection of usable characters for determining the possible cryptic *Stenotaenia linearis* taxa. However, it is not feasible to have such morphological studies as part of a large barcoding project like GBOL.

### Taxonomic implications

Our analysis shows the importance of COI barcode data in the detection of taxonomic problems inside the centipede order Geophilomorpha. However, it also illustrates that barcode data alone does not clarify taxonomic problems. Only a thorough morphological study of the *Stenotaenia* species, including the types, plus the addition of nuclear markers, may be able to solve the complex picture of this genus.

As a result of the voucher-based barcoding effort, all analyzed specimens, and even their DNA extracts, are available for loan and should be incorporated into any future study of *Stenotaenia*.

## Supplementary materials

Supplementary material 1 Table. Estimates of Evolutionary Divergence between Sequences Data type: Measurement Explanation note: The number of base differences per site from between sequences are shown. The analysis involved 45 nucleotide sequences. Codon positions included were 1st+2nd+3rd+Noncoding. All ambiguous positions were removed for each sequence pair. There were a total of 658 positions in the final dataset. Evolutionary analyses were conducted in MEGA6. This dataset is made available under the Open Database License (http://opendatacommons.org/licenses/odbl/1.0/). The Open Database License (ODbL) is a license agreement intended to allow users to freely share, modify, and use this Dataset while maintaining this same freedom for others, provided that the original source and author(s) are credited.Thomas Wesener, Karin Voigtländer, Peter Decker, Jan Philip Oeyen, Jörg Spelda, Norman Lindner
